# A Patient-Based Needs Assessment for Living Well with Parkinson Disease: Implementation via Nominal Group Technique

**DOI:** 10.1155/2013/974964

**Published:** 2013-02-17

**Authors:** Galit Kleiner-Fisman, Pearl Gryfe, Gary Naglie

**Affiliations:** ^1^ATC and Jeff and Diane Ross Movement Disorders Clinic, Baycrest Hospital, University of Toronto, 3560 Bathurst Street, Toronto, ON, Canada M6A 2E1; ^2^Baycrest Hospital, University of Toronto, 3560 Bathurst Street, Toronto, ON, Canada M6A 2E1

## Abstract

*Background*. Parkinson's disease (PD) is a neurodegenerative condition with complex subtleties, making it challenging for physicians to fully inform their patients. Given that approximately 50% of Americans access the Internet for health information, the development of a multimedia, web-based application emphasizing targeted needs of people with Parkinson's disease (PwP) has the potential to change patient's lives. *Objectives*. To determine what information PwP perceive could enhance their quality of life. *Methods*. Group sessions utilizing nominal group technique (NGT) were conducted. Participants were asked “what information do you want to know about that would help you live well with PD?” Silent generation of ideas preceded discussion followed by anonymous ranking of items. A “summary score” (sum of rank × frequency) was calculated. *Results*. 36 individual items were collapsed into 9 categories. Coping with emotions, changing relationships, and social implications of PD were ranked as most important. Financial supports and skills for self-advocacy were also highly ranked. *Conclusions*. Qualitative research methodology was utilized to determine the unmet needs of PwP. Results of this survey will inform the development of a patient-oriented, online resource, the goal will be to provide information and strategies to improve symptom management, reduce disability and address all relevant concerns important to those affected by PD.

## 1. Introduction

The incidence of Parkinson's disease (PD) increases with advancing age, and incidence exceeds 1% after age 65, and 3% after age 80 [1]. The prevalence of PD in those over 80 is projected to double over the next 30 years (from 11.6% to 23.6%) as life expectancy in the elderly increases. In 1998, an estimate of annual PD-related costs in Canada exceeded $560 million, with 70% of these costs related to long-term disability [2]. As with prevalence, the socioeconomic impact of PD is projected to increase dramatically; in Canada, health cost inflation and an aging population are likely to result in costs of almost $1 billion annually in the coming decades. Costs related to lost income for both people living with PD (PwP) and caregivers, and intangible costs associated with emotional anguish, are likely also large [3–7]. The progressive nature of PD symptoms results in profound reduction in quality of life that in some cases can be rated worse than death (based on commonly used health utility measures [8]).

Evidence, knowledge, and strategies to delay functional decline in PD *do* exist. A recent study showed that disease-specific, seminar-based knowledge provision improved mood and overall health-related quality of life in PwP and in their caregivers [9]. Another study found that a multidisciplinary intervention program including speech therapy, occupational therapy and physical therapy, improved quality of life [10]. Other studies have confirmed that people with access to educational programs, knowledgeable specialists, and allied health professionals have improvements in quality of care and increased satisfaction with care [1, 11–13].

Unfortunately, the high prevalence of PD and limitations in access to PD-focused specialist and multidisciplinary care mean that many PwP fail to benefit fully from existing knowledge. As medical care models become increasingly patient centric, emphasis on provision of up-to-date, accurate, and comprehensible information on PD to facilitate active participation in decision-making will be central to meeting patient needs.

Over the past decade, the Internet has emerged as a medium with great potential to disseminate both medical information and medical misinformation [14]. Given challenges in providing adequate access to PD-specialty care, and the importance of communicating disease-specific information to patients to improve quality of life and empower people, the Internet represents an attractive platform for dissemination of PD-related information. Older individuals (including those aged > 65) *do* use the Internet as a primary source of health information [15] though not all have access to a computer. However, of the estimated 4.6 million webpages currently devoted to PD, only 1% of the top 100 of these have been judged to be comprehensible by the average person [16]. To our knowledge, none are created by healthcare practitioners with direct practical experience of the needs and sensitivity to the specific challenges of PwP.

Our aim is to develop a PD-specific website that incorporates the input of those PwP, and input from a multidisciplinary clinical team with extensive experience in treating PwP and their families. The website is intended to be a multimedia platform that serves as a comprehensive and reliable source of information on self-management and maintenance of independence. In order to create this website in a manner that addresses the perceived needs of patients themselves, and to do so in a rigorous and reproducible manner, we conducted a structured needs assessment of PwP using nominal group technique [17]. The advantage of this approach is that it provides a structure whereby groups of individuals can present disparate views on a subject, with all views incorporated into a rank-ordering process [17–19]. We used NGT to identify and organize broad “domains” of identified informational needs, and to generate a semiquantitative rank ordering of informational resources that PwP themselves perceive to be most in need for “living well” with PD.

## 2. Methods

### 2.1. PwP/Caregiver Participants

PwP were invited to share their lived experience in multiple group discussions. The Parkinson Society of Canada (PSC) (Toronto, ON, Canada) sent out mailings advertising the project. Interested individuals contacted our Center and were invited to take part in focus groups. Groups were assembled to include a heterogeneous population of PwP to include those with mild symptoms and those with more advanced disease. Only those fluent in English were included. Individuals with communication or cognitive impairment due to PD were included with their spouses as unique PwP-caregiver dyads (2 pairs). As we were interested in the patient perspective, for the patient-caregiver dyads, we asked the caregiver to facilitate the PwPs' responses rather than provide their own perspectives.

 The Ethics Review Board (ERB) of Baycrest Health Sciences approved the study. All participants including patients and caregivers signed the ERB-approved, written informed consent form before participation.

### 2.2. Group Discussion Using Nominal Group Technique

We used nominal group technique (NGT) [17, 19–21] to facilitate the identification of informational gaps that PwP perceived in their current care or in care previously received, or that PwP thought would help them to live the “best possible life” with PD. NGT is particularly useful when there is an interest in rapidly identifying and rank-ordering multiple potential priorities, rather than deciding on a single most important item via majority rule, when some individuals in a group may be less likely to share opinions and thoughts than others, and (as here) where there is the potential for a power imbalance between group members and the facilitator due to perceived differences in subject-specific knowledge (as might occur when the facilitator is a specialist physician and participants are patients).

NGT was conducted with three unique groups of PwP (or PwP-caregiver dyads) with 6–8 participants/group, in keeping with standard methods for NGT. This small number of participants allows for maximal and in-depth contribution from all members. Three group sessions were deemed adequate as data saturation was encountered by the 3rd group and no new themes emerged [22].

Briefly, each session began with an orientation by the facilitator (G. Kleiner-Fisman), and with an explanation of the flow of the meeting; this was followed by silent written generation of ideas, with participants providing initial written answers to the question

*“What information is missing from your healthcare experience or has been missing in the past that would help you' live well with PD?”*

Following silent generation of ideas, participants engaged in “idea sharing,” with responses recorded on a flip chart. The facilitator ensured that every participant offered a single and unique initial response. Subsequently, participants engaged in a facilitated discussion, where they sought clarity on ideas proposed by others, with the facilitator striving to maintain a neutral and non-critical tone to the discussion, and ensuring that all participants were heard. Round-robin idea sharing and discussion continued until all unique responses from all participants were exhausted. A final list of items for each session was compiled and presented to the respective group. Wording of each item was discussed with the group until consensus was achieved and the group discussion ended. 

Lastly, participants voted on and ranked ideas produced during earlier phases of this process. Participants were asked to anonymously assign an importance score from one to seven for each item in order of their priority (1 = lowest priority; 7 = highest priority) [17]. After all group sessions had been completed, the investigators created a final composite list of all items and rankings from all three groups. Redundant items were removed. Preliminary categories were presented to participants and further refined until consensus regarding logical groupingwas reached. Summary scores for each item were calculated (frequency of responses × [sum of importance scores/item]) [23]; the highest score was arbitrarily assigned a value of 100 points, and the scores were rescaled to a 0–100 scale by dividing each by the highest score and multiplying by 100.

## 3. Results

### 3.1. Focus Group Participants

A total of thirty individuals contacted our Center to inquire about the focus groups. Of these, twelve were unable to participate due to a lack of English fluency (3), transportation difficulties (5), and being unwell on the day of the session (4). Seventeen PwPs (15 PwPs and 2 PwP-caregiver dyads (4)) were included in 3 separate sessions. Participant characteristics are shown in Table 1. Most (72%) received care from movement disorders specialists; the remainder of the group received care from general neurologists.

### 3.2. Identified Needs and Rankings

**Figure 1:**
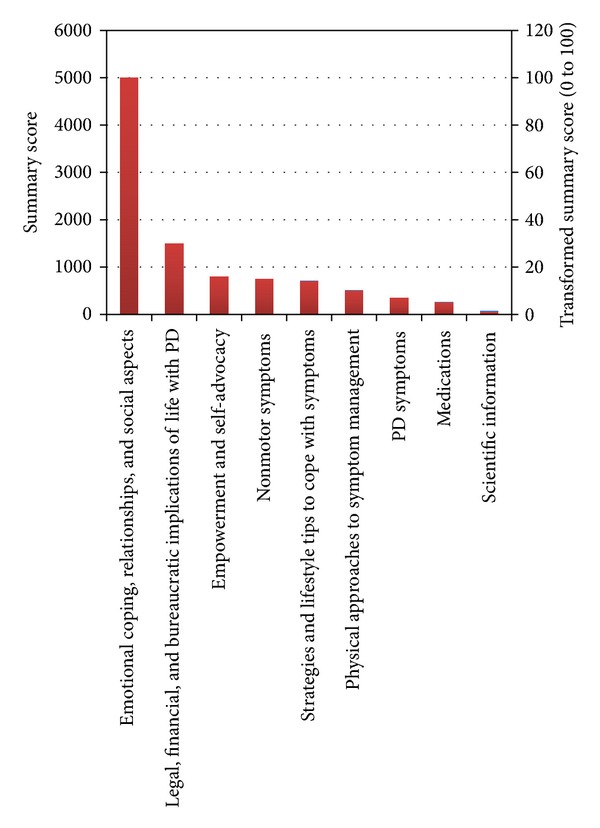
Summary scores for informational categories identified by patients as missing from their care. Raw scores are plotted on the left *Y*-axis; rescaled (0–100 scale) are plotted on the right *Y*-axis.

The themes that emerged from all 3 PwP/caregiver group sessions were compiled and condensed into a single list of 36 items (summarized below) that were further collapsed into 9 broad categories ranked according to “summary score” (Table 2). Categories were ranked by participants in descending order, based on the impact on health and functioning. Summary scores are presented graphically in Figure 1. The highest score was assigned to issues related to emotional coping, relationships, and social aspects of life with PD. Legal, financial, and bureaucratic implications of life with PD received the second highest score. Issues related to physical and symptomatic aspects of life with PD were ranked less highly. 

## 4. Discussion

Parkinson's disease is a common and costly neurodegenerative disease in older adults, and its prevalence is increasing. As such, identifying the means to improve the quality of life of PwP will become ever more important in the coming decades. Although patient-centered care is often endorsed in theory, practical realization of care that is patient-centered will not occur unless the views, opinions, beliefs, and attitudes of patients themselves are considered as a key component of therapeutic encounters. Patient centeredness is defined by the Institute of Medicine as “providing care that is respectful of and responsive to individual patient preferences, needs, and values, and that ensuring patient values guide all clinical decisions” [24], and is increasingly considered a standard to which healthcare providers are held by third-party funders [25].

In the present study, we used qualitative and semiquantitative methodologies to gain insights into the educational and informational needs of PwP. While our specific aim in this effort was to identify priority content areas for a patient-centered website, our use of a rigorous qualitative approach provides important insights into the informational gaps that PwP perceive as being detrimental to their health and quality of life in living with PD.

Perhaps most noteworthy is our finding that gaps identified most commonly, and weighted most heavily, by PwP, were not those that involved disease symptomatology and treatment, but rather gaps related to the emotional and social challenges experienced by PwP, and the legal and financial difficulties encountered by those living with the disease. Though it is possible that some items could have been classified into alternate categories, this does not dilute the overall message that psychosocial issues are dominant in the minds of our participants. Though psychosocial issues related to coping with PD have been recognized by some investigators as contributors to reduced quality of life in PD [26, 27], healthcare provider focus is generally on symptom management and medication adjustment as reflected in professional guidelines [28]. PD guidelines do not explicitly address psychosocial issues. Many neurologists would argue that, given the extensive knowledge base required for optimal medical management, it would be impossible for neurologists to master the nuances of such dimensions of the disease. We agree, and indeed this reinforces the importance of both multidisciplinary care provision for PwP (with teams that include mental health professionals and social workers) and also, as noted above, this underlines the importance of providing patients with other sources of such critical information, such as via the Internet.

As with psychosocial issues, financial issues in PD emerged as a major concern in our patient population. Our study participants identified financial considerations as a significant preoccupation and source of anxiety. As noted above, indirect costs of PD may exceed direct healthcare costs, with the largest component cost being income loss related to early retirement; the risk of early retirement was 5 times greater in PwP than in age-matched controls [29]. Web-based information provision is an optimal method of disseminating this form of information in conjunction with access to social services.

We note that participants emphasized the importance of being empowered to make educated decisions regarding their own health. Empowerment in the context of “patient-centeredness” is increasingly being recognized as an important element in optimizing quality of care [30]. Understanding mechanism of disease and how symptoms can present (both motor and nonmotor), natural history of the condition, treatment options, including risks and benefits, and troubleshooting problems, is critical information that a person must possess in realizing the goal of empowerment to make informed medical decisions. A multimedia web-based application may be an ideal medium in which to disseminate such complex information.

Like any clinical study ours has limitations, most notably the generalizability of findings from our focus groups. Participants were relatively young, highly educated, very motivated, and had limited disability, relative to the PwP as a whole. Given the fact that the participants in these groups were relatively informed, it is likely that PwP that do not have access to movement disorders specialists, would have different priorities and would more likely be focused on basic information regarding symptoms and medications. Similarly, concerns that may emerge with more debilitated populations include behavioral and psychiatric problems, alternative living arrangements, legal issues related to assigning power of attorney and end of life-care. Though not identified by our cohort, these subjects would be reasonable to additionally address as content in a web-site.

PwP with lower levels of formal education and knowledge regarding PD and family members and caregivers of patients with later stages of PD (who cannot themselves participate) represent important future targets for our group. Nonetheless, the priorities identified in our focus groups are completely in keeping with the WHO responsiveness model of care in health care [31] and other studies assessing patient perceptions regarding unmet needs [30, 32]. Van der Eijk et al. [30] recently performed a survey to assess perception of “unmet needs” of PD patients in The Netherlands; like our cohort, PwP and informal caregivers desired more emotional support from healthcare professionals and wanted more active involvement in clinical decision making though felt they lacked the necessary knowledge. Grosset et al., in a study looking at factors related to satisfaction in care found that patient involvement in therapy decisions correlated with satisfaction in care. Satisfaction with care also correlated with medication compliance and quality of life. While these studies were not intended to generate domains for web-based content, they nonetheless provide a degree of reassurance regarding the generalizability of our findings.

## 5. Conclusions

We used nominal group technique to identify informational gaps perceived by PwP, which might be improved through the creation of a web-based utility. In a group of relatively young and educated PwP, concerns focused particularly on psychosocial and financial challenges associated with PD. The PwP-centric nature of this exercise was key, as it is imperative that PwP are included in the process of developing patient-directed knowledge translation materials. This will allow us to create a PwP-oriented multimedia online resource that enhances care, provides critical information, and complements services provided by traditional healthcare providers.

## Figures and Tables

**Table 1:** Demographic characteristics of PwP participating in focus group (*n* = 17, 2 caregivers not included).

Characteristic	Mean (%)	SD or CI
Male = 39%		
Age (43–84)	64.4	9.4
Disease duration (1–17 yrs)	8.8	4.7
Schwab and England ON (0–100)	81.3	54.4–96.0
Marital status		
Married	67.0	
Divorced	17.0	
Single	11.0	
Widowed	5.0	
Level of education completed		
Post graduate	33.0	
Undergraduate	28.0	
College diploma	22.0	
High school	17.0	
Employment status		
Retired	73.0	
Disability pension	17.0	
Short-term disability	5.0	
Employed	5.0	
Living arrangements		
With spouse	67.0	
Alone	23.0	
With children	5.0	
With parent	5.0	

**Table 2:** Results of needs assessment from nominal group technique regarding what PD patients believe to be missing from their healthcare and educational experience.

Rank	Categories	Items or ideas included	Frequency	Importance score
(1)	Emotional coping, relationships, and social aspects	(i) Dealing with fear about the future. (ii) Coping strategies. (iii) Coming to terms with living with a chronic illness. (iv) Challenges for marital relationship (including helping unaffected spouses come to terms with living with a chronically ill individual). (v) Availability of programs that promote a sense of wellbeing in dealing with disease.	34	143

(2)	Legal, financial, and bureaucratic implications of life with PD	(i) Legal implications of living with PD (e.g., maintenance of driver's license; proxy decision making). (ii) Financial implications of living with PD (e.g., public financial assistance and subsidies; financial and estate planning). (iii) Transportation. (iv) Managing government paperwork. (v) Where to find local resources for people with disabilities. (vi) Health-care system interactions.	19	76

(3)	Empowerment and self-advocacy	(i) Managing one's own expectations. (ii) Managing expectations of family and friends. (iii) Strategies to ensure assertive and informed participation in health-related decisions.	16	50

(4)	Nonmotor symptoms	(i) Sleep difficulties. (ii) Bowel, and bladder dysfunction. (iii) Mood changes. (iv) Difficulties with cognition. (v) Holistic approaches to managing nonmotor symptoms.	11	65

(5)	Strategies and lifestyle tips to cope with symptoms	(i) Practical solutions for common symptoms experienced in PD. (ii) Use of adaptive technologies to facilitate activities of daily living and home safety.	15	47

(6)	Physical approaches to symptom management	(i) Role of exercise in PD management. (ii) Information on accessible home activity programs. (iii) Management of daily physical challenges (e.g., rolling over in bed or getting out of chair).	11	46

(7)	PD symptoms	(i) Definition of “symptoms of parkinsonism.” (ii) Differentiation of PD symptoms from medication side-effects.	10	35

(8)	Medications	(i) Correct use of medications (e.g., timing, quantity). (ii) Common drug side-effects and their management. (iii) Relationship between diet and medications.	8	33

(9)	Scientific information	(i) Genetic basis of PD and heritability. (ii) Current promising areas of research. (iii) Etiology of PD.	5	15

Though there were 16 respondents, the frequency is > than 16 due to the fact that multiple responses from individual participants were collapsed into single categories.
